# Editorial: Application of gene editing in neurodegenerative diseases, volume II

**DOI:** 10.3389/fnins.2023.1305302

**Published:** 2023-10-31

**Authors:** Fangfang Qi, Xingshun Xu, Xing Guo, Junhong Luo, Yujing Li, Sen Yan

**Affiliations:** ^1^Department of Anatomy and Physiology, Guangdong Province Key Laboratory of Brain Function and Disease, Advanced Medical Technology Center, The First Affiliated Hospital, Zhongshan School of Medicine, Sun Yat-sen University, Guangzhou, China; ^2^Department of Neurology, Mayo Clinic, Rochester, MN, United States; ^3^The Institute of Neuroscience, Soochow University, Suzhou, China; ^4^Department of Neurobiology, School of Basic Medical Sciences, Nanjing Medical University, Nanjing, Jiangsu, China; ^5^Department of Systems Biomedical Sciences, School of Medicine, Jinan University, Guangzhou, China; ^6^Department of Human Genetics, Emory University, Atlanta, GA, United States; ^7^Guangdong Key Laboratory of Non-human Primate Research, Guangdong-Hongkong-Macau Institute of CNS Regeneration, Jinan University, Guangzhou, China

**Keywords:** gene editing, neurodegenerative diseases, adeno-associated virus (AAV), animal model, CRISPR/Cas9

Neurodegenerative disorders (NDs) are a broad category of ailments caused by progressive damage to cells and the nervous system that affect millions of individuals worldwide. The most prevalent neurodegenerative disorders include Alzheimer's disease (AD), Parkinson's disease (PD), Huntington's disease (HD), Frontotemporal dementia (FTD), and Amyotrophic lateral sclerosis (ALS).

The pathophysiology of NDs is known to be linked with gene mutations, including (1) presenilin (*PSEN*) and amyloid beta precursor protein (*APP*) mutations in AD; (2) mutations in *PARK* genes such as *FBXO7, ATP13A2, SYNJ1, PLA2G6, DNAJC6, PINK1*, and *PRKN* in PD, which participated in neuronal developmental processes; (3) mutant huntingtin gene (*HTT*) in HD; (4) *TARDBP* and *Stmn2* in FTD; (5) mutations in *C9orf72*, superoxide dismutase (*SOD1*), *TARDBP, Stmn2*, and fused in sarcoma (*FUS)* gene in ALS, and so on.

CRISPR-Cas9 has shown considerable potential in treating neurodegenerative diseases. The development of the clustered regularly interspaced short palindromic repeats (CRISPR)-Cas9 system has revolutionized the area of gene editing, which has been widely used in numerous cell types and creatures for efficient gene disruption and gene alteration, both *in vitro* and *in vivo*. Recent studies have revealed new insights into the progress of CRISPR/Cas9-mediated genome editing and its application to neurodegenerative illnesses. As a result, genome editing and gene transfer remain viable techniques for the potential management of NDs for correcting gene mutations.

This Research topic was primarily concerned with discovering new cellular pathological traits based on gene-edited ND models, as well as current breakthroughs in ND model construction and prospective therapeutic techniques using CRISPR-Cas9 systems. This topic presents an overview of current research on the development of novel PD- and ALS-related experimental models, including cellular models, small animal models, and large mammalian models, as well as the use of CRISPR/Cas9 technology. The possible translation of ND models to future clinical therapies was a prominent theme that emerged.

Mutant *HTT* can both affect mature neuronal functions in adulthood and neuronal development in the embryonic period, and early life for HD. Therefore, HD has been considered a neuronal development-related disease. However, less is known about whether mutant *HTT* affects glial development, thereby contributing to early neuronal development defects. In this Research Topic, Yang et al. reported mutant *HTT* did not affect astrocyte and oligodendrocyte development but impaired myelination in the early disease stage using HD knock-in mice that express full-length mutant *HTT*. This study suggested that cytoplasmic mutant *HTT* is more likely to damage neuronal functions than glial cells in an HD knock-in mouse model. These findings add to our understanding of myelination loss and have therapeutic potential for preventing HD neuropathology.

PD can be divided into sporadic and familial types, with the latter accounting for 10–15%. Familial PD is usually caused by mutations in PD-related genes, including *SNCA, Parkin, PINK, DJ-1, LRRK, ATP13A*, and so on. Therefore, CRISPR Cas9 and related gene editing technology have enabled humans to explore the relationship between genes and diseases more precisely. In this Research Topic, Qu et al. reviewed the details and development of CRISPR Cas9, the construction of PD-related animal models, and the therapeutic methods for PD via CRISPR Cas9 and related technologies will be provided. Notably, this review summarized multiple experimental models, including iPSC cells of patients with *LRRK2* p.G2019S and *PARK2* gene mutations, human dopaminergic SH-SY5Y cell lines with *UQCRC1*gene mutation, zebrafish model with double knockout of *mcu* and *pink1, Atp13a2*^−/−^ zebrafish, *Vps35*- or *Cdk5*-deficient mouse models, as well as triple knock-out (*Parkin, Dj-1*, and *Pink1*) Bama miniature pigs, *Pink1* and *Dj-1* deficient monkey model. Furthermore, the application and effects of CRISPR Cas9 in treating PD were also summarized by the authors. However, it is important to note that improving the CRISPR-Cas9 system's delivery efficiency and lowering the side effects on the brain in optimized stereotactic injection studies will be critical for its use in gene editing for PD treatment.

Amyotrophic lateral sclerosis (ALS) is another progressive neurodegenerative disease, that affects motor neurons (MNs) in the spinal cord, brainstem, and motor cortex. As previously stated, the majority of ALS cases are sporadic and have an unknown origin, however, roughly 10% of individuals have a family history of the disease, strongly implying a genetic component. Over 50 genes have been identified as ALS-associated genes, including *SOD1, C9orf72, TARDBP*, and *FUS*. Therefore, exploration of more effective mutant gene-related pathophysiology of ALS is better for developing prospective therapeutic targets.

Similarly, different disease models were built using gene-editing technology, such as HT22, iPSCs, BV2, Neuro 2a, NSC-34, hESC, and HeLa cell lines for cell models, zebrafish model with *TP73* deficient, *C. elegans* model, deficient mouse models (*CREST*-, C9orf72-deficient or *Q394X* knockin mice), as well as CRISPR/Cas9-targeted large animals (pig or monkey). Furthermore, three main gene targets, *SOD1 C9ORF72, FUS* and *TARDBP*-based application of the CRISPR/Cas9 technology for ALS therapy were also reviewed by Shi et al. in this Research Topic.

In addition, the application of gene therapy is not only limited to brain diseases but is also are found in retinal neurodegenerative diseases such as glaucoma and optic nerve injury (ONI). As we know, optic nerve injury is generally considered irreversible. Thus, using the CRISPR system or adeno-associated virus (AAV), brain-derived neurotrophic factor (BDNF), ciliary neurotrophic factor (CNTF), phosphatase-tensin homolog (PTEN), suppressor of cytokine signal transduction 3 (SOCS3), histone acetyltransferases (HATs) were edited to explore their roles in ONI protection. In this Research Topic, Xu et al. reviewed the research progress in gene therapy for optic nerve injury, which is characterized by the loss of retinal ganglion cells (RGCs) and axons, to protect both RGCs and axons.

Together, this Research Topic revealed novel pathogenic pathways, novel therapeutic targets by gene-editing, breakthroughs in experimental models and preclinical research, and clinical treatment problems in the field of neurodegenerative disorders. We believe that all of these projects will contribute to the advancement of basic research and clinical applications in the future.

## Author contributions

FQ and SY drafted the editorial. FQ and SY revised the editorial with contributions from all authors. All authors approved the final version.

